# Exploring the Effect of Asymmetric Mitochondrial DNA Introgression on Estimating Niche Divergence in Morphologically Cryptic Species

**DOI:** 10.1371/journal.pone.0095504

**Published:** 2014-04-17

**Authors:** Ben Wielstra, Jan W. Arntzen

**Affiliations:** 1 Naturalis Biodiversity Center, Leiden, The Netherlands; 2 Department of Animal and Plant Sciences, University of Sheffield, Sheffield, United Kingdom; Onderstepoort Veterinary Institute, South Africa

## Abstract

If potential morphologically cryptic species, identified based on differentiated mitochondrial DNA, express ecological divergence, this increases support for their treatment as distinct species. However, mitochondrial DNA introgression hampers the correct estimation of ecological divergence. We test the hypothesis that estimated niche divergence differs when considering nuclear DNA composition or mitochondrial DNA type as representing the true species range. We use empirical data of two crested newt species (Amphibia: *Triturus*) which possess introgressed mitochondrial DNA from a third species in part of their ranges. We analyze the data in environmental space by determining Fisher distances in a principal component analysis and in geographical space by determining geographical overlap of species distribution models. We find that under mtDNA guidance in one of the two study cases niche divergence is overestimated, whereas in the other it is underestimated. In the light of our results we discuss the role of estimated niche divergence in species delineation.

## Introduction

Potential morphologically cryptic candidate species are often first identified based on diverged mitochondrial DNA (mtDNA) [1], especially with the increased reliance on mtDNA barcoding as a tool for species identification and biodiversity estimation [2]. Because ecological divergence can promote speciation [3]–[5], a handle on potential species status may be provided if mtDNA divergence is paralleled by niche divergence [6], [7]. However, mtDNA does not always properly reflect species boundaries as defined by nuclear DNA (nuDNA) and asymmetric introgression of mtDNA is commonplace [8]. During species displacement accompanied by hybridization, mtDNA is prone to introgress from the native, common species to the invading and locally rare species, due to the demographical imbalance at the hybrid zone [9], [10]. Alternatively, positive selection could cause mtDNA to be pulled into the range of another species [11]. This raises the question of how asymmetric mtDNA introgression would affect the estimation of niche divergence in diagnosing species.

**Figure 1:**
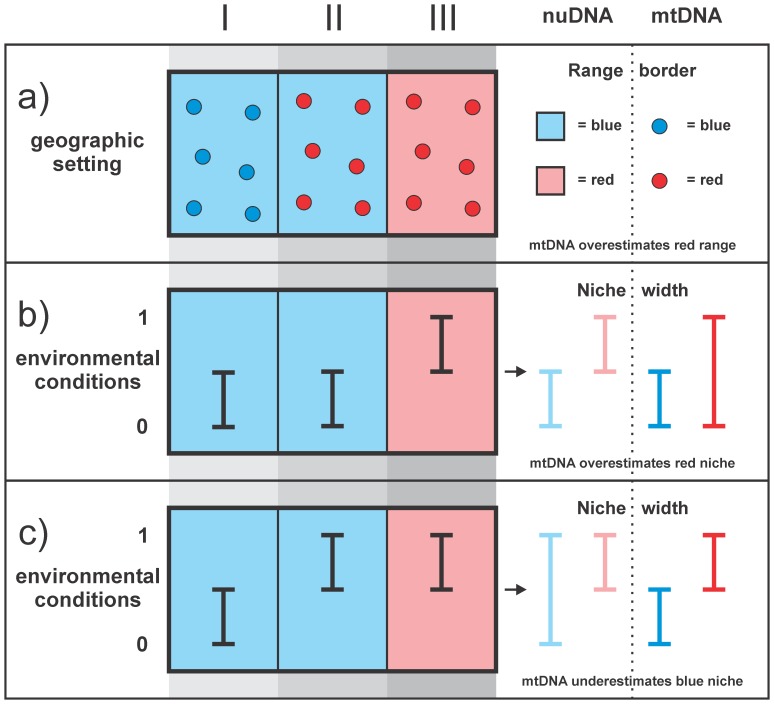
The potential effect of asymmetric mtDNA introgression on estimating the niche divergence of species. Schematic with two species shown in blue and in red, mtDNA shown by hard colors inside symbols, nuDNA shown by soft colors in the background, and range sections shown as I, II and III. In (a) the two species possess their own mtDNA in range sections I and III whereas in range section II the blue species possesses mtDNA of the red species. Treating mtDNA as species diagnostic overestimates the range of the red species. The other two panels illustrate environmental conditions under which species niche divergences are flawed by following the mtDNA guidance. Note the difference in occupied niche space in range section II that under scenario (b) results in an underestimate of the true species niche divergence and yields an overestimate in scenario (c).

When interpreting mtDNA divergence as coinciding with species ranges, asymmetrically introgressed mtDNA would distort niche divergence estimates, by wrongfully excluding a locality from one lineage and including it with another (Fig. 1a). Two contrasting effects might result. On the one hand, niche divergence between the two species could be underestimated, because part of the niche space occupied by species 1 is erroneously interpreted as also being occupied by species 2 (Fig. 1b). On the other hand, niche divergence between the two species could be overestimated, because part of the niche space occupied by both species is erroneously interpreted as being only occupied by species 2 (Fig. 1c). Here we explore these potential effects, using empirical data of crested newts (Amphibia: *Triturus*), distributed in SE Europe and adjacent Asia.

### Crested Newt Case Studies

**Figure 2:**
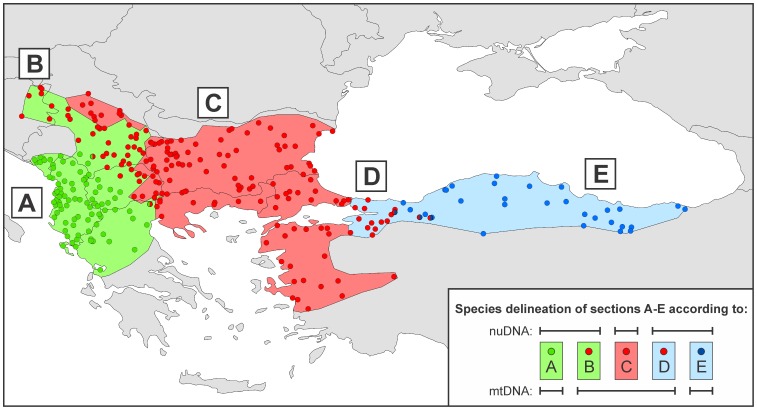
Map showing ranges and localities of three *Triturus* species in SE Europe and adjacent Asia. The ranges shown by light shading reflect nuDNA composition, whereas the localities shown by dots reflect the observed mtDNA type. In part of their ranges both *T. macedonicus* and the candidate species possess mtDNA derived from *T. ivanbureschi* [22], [36]. Five range sections are recognized: range section **A** where *T. macedonicus* possesses species-specific mtDNA, range section **B** where *T. macedonicus* possesses introgressed mtDNA, range section **C** where *T. ivanbureschi* possesses species-specific mtDNA, range section **D** where the candidate species possesses introgressed mtDNA, and range section **E** where the candidate species possesses species-specific mtDNA.

MtDNA introgression is regularly observed at crested newt contact zones [12]. We here present two cases, at different stages of taxonomic development. The first case concerns *T. macedonicus*. *Triturus macedonicus* was revealed as an allopatric sister lineage of *T. carnifex* based on mtDNA and morphology [13], [14] and is currently recognized as a distinct species based on nuDNA divergence [15]. Here asymmetric introgression of mtDNA, derived from *T. ivanbureschi*, is found over a considerable part of the Balkan Peninsula (Fig. 2). Morphological criteria such as the number of rib-bearing vertebrae can be used to distinguish *T. macedonicus* from *T. ivanbureschi*, but species identification in the field is not always straightforward [14], [16], [17].

The second case concerns an as yet undescribed taxon (hereafter referred to as “candidate species”) [18]. The candidate species was first identified as distinct from *T. ivanbureschi* based on diverged mtDNA [13], [19], [20], subsequently found to occupy different environmental space [21], and eventually suggested to represent a distinct nuDNA gene pool as well [22]. The distribution of nuDNA and mtDNA does not fully overlap: the candidate species possesses mtDNA derived from *T. ivanbureschi* in NW Asiatic Turkey (Fig. 2). There are as yet no documented morphological criteria to distinguish the candidate species from *T. ivanbureschi* [16], [17], [23].

We test the hypothesis that estimated niche divergence based on the true species boundary (i.e. based on nuDNA) differs from that based on only mtDNA (misrepresenting the true species due to geographically asymmetric introgression) and in the light of our results we discuss the role of estimated niche divergence in species delineation.

## Methods

### Distribution and Environmental Data

We used ref. [12] as a base for our locality dataset. We incorporated 18 newly identified localities, three of which replace localities with a less accurate provenance from ref. [12]. We excluded 13 localities where only genetically admixed individuals were found based on 52 nuDNA markers (ref. [24]; Wielstra et al. in prep.) and two further localities for which no nuDNA could be consulted but which were positioned close to admixed populations. The dataset encompassed 300 localities and was partitioned based on nuDNA and mtDNA as follows: *T. macedonicus* 127 nuDNA and 89 mtDNA localities, *T. ivanbureschi* 127 nuDNA and 183 mtDNA localities and the candidate species 45 nuDNA and 32 mtDNA localities. Five range sections were recognized based on the combination of nuDNA composition and mtDNA type as follows: range section **A** (*T. macedonicus* with species-specific mtDNA) with 89 localities over c. 85,200 km^2^, range section **B** (*T. macedonicus* with introgressed mtDNA) with 40 localities over c. 49,200 km^2^, range section **C** (*T. ivanbureschi* with species-specific mtDNA) with 127 localities over c. 224,300 km^2^, range section **D** (candidate species with introgressed mtDNA) with 16 localities over c. 16,700 km^2^ and range section **E** (candidate species with species-specific mtDNA) with 32 localities over c. 100,000 km^2^. Species-specific and introgressed mtDNA were found in syntopy in two *T. macedonicus* and three candidate species localities. For details see Fig. 2 and Dataset S1. For ease of communication we use range section names **A**–**E** throughout the Methods and Results.

For environmental data layers, we used bioclimatic variables at 2.5 arcminute resolution (c. 5×5 km) available from the WorldClim database 1.4 [25]. To obtain realistic and transferable models, it is recommended to mirror the physiological limitations of the study species and minimize the effects of multicollinearity among data layers [26]–[29]. Crested newt species differ in the length of their annual aquatic period [16], [19]. Therefore, we included a set of layers that likely reflects the availability of water bodies during the breeding season, i.e. seasonal variation in evaporation and precipitation: bio10 = mean temperature of warmest quarter, bio11 = mean temperature of coldest quarter, bio15 = precipitation seasonality, bio16 = precipitation of wettest quarter, and bio17 = precipitation of driest quarter. These layers show a Pearson correlation <0.7. This selection is identical to the one used by us before [12], [21].

### Niche Divergence in Environmental Space

Environmental values of the bioclim layers corresponding to each crested newt locality were extracted in ArcGIS (www.esri.com). The data were standardized to a mean of zero and a standard deviation of one to eliminate measurement-scale effects. Niche divergence in multidimensional space derived from the principal components axes were quantified with Fisher distances under default settings with XLSTAT 2013 (www.xlstat.com) and used to test the following null hypotheses (see also Fig. 2): 1) the environmental conditions experienced by species are not significantly different, i.e. Fisher distances are not significantly different from zero, if species are delineated based upon either mtDNA or nuDNA (**A+B** vs. **A** for *T. macedonicus*, **C** vs. **B+C+D** for *T. ivanbureschi* and **D+E** vs. **E** for the candidate species); 2) the environmental conditions of the areas with species-specific and introgressed mtDNA are not significantly different, i.e. Fisher distances are not significantly different from zero, within species (**A** vs. **B** for *T. macedonicus*, **D** vs. **E** for the candidate species); and 3) the environmental conditions in the area where *T. macedonicus* and the candidate species possess introgressed mtDNA are not *more* dissimilar (i.e. Fisher distances are relatively larger) to those encountered in the range of the mtDNA ‘donor’ *T. ivanbureschi* than those encountered in the area where species possess species-specific mtDNA (**A** vs. **C** compared to **B** vs. **C** for *T. macedonicus*, **C** vs. **D** compared to **C** vs. **E** for the candidate species).

### Niche Divergence in Geographical Space

We used Maxent 3.2.1 [30] to construct species distribution models for species as identified based on nuDNA (**A+B** for *T. macedonicus*, **C** for *T. ivanbureschi* and **D+E** for the candidate species) and based on mtDNA (**A** for *T. macedonicus*, **B+C+D** for *T. ivanbureschi* and **E** for the candidate species). We constrained the background (i.e. the area from which pseudo-absence data are drawn) to a 200 km buffer zone around known *Triturus* localities (see ref. [12]), the rationale being that excluding highly distinct environmental information, occurring outside the area of interest, prevents inflation of model performance and predicted suitable area [31]. We tested whether any of the species distribution models performed statistically significantly better than random, following the null model approach of Raes and ter Steege [32].

Geographical overlap of the species distribution models was quantified with Schoener’s *D* in ENMTools 1.3 [33], [34] as *D* was found to perform best in a comparison of various commonly used metrics [35]. *D* values range from 0 (no overlap) to 1 (complete overlap). Because inclusion of a high proportion of grid cells with low occurrence probabilities – presumably representing background noise – may bias assessments of niche overlap [35] we restricted the geographical area for which niche overlap was determined to that over which the three species are distributed (cf. Fig. 2).

We determined *D* values to test the following null hypotheses (see also Fig. 2): 4) models for pairs of species as identified with nuDNA and mtDNA, **A** vs. **A+B** for *T. macedonicus*, **C** vs. **B+C+D** for *T. ivanbureschi* and **E** vs. **D+E** for the candidate species, show a similar degree of geographical overlap, i.e. *D* values are not significantly different, and 5) models for the two pairs of species that show mtDNA introgression show a similar degree of geographical overlap, i.e. *D* values are not significantly different, when identified based on nuDNA or mtDNA, i.e. the two pairwise comparisons **A+B** vs. **C** compared to **A** vs. **B+C+D**, for *T. macedonicus* vs. *T. ivanbureschi*, and **C** vs. **D+E** compared to **B+C+D** vs. **E**, for *T. ivanbureschi* vs. the candidate species. The test values were obtained by running Maxent ten times, under the random subsampling of 70% of the locality data (the ‘subsample’ replicated run type with 30% of locality data used as ‘random test percentage’), yielding 10×10 = 100 semi-replicate *D* values for each of the five pairwise comparisons. The sets of *D* values were tested with ANOVA and interpreted through a Tukey’s HSD *a posteriori* test in SPSS 16.

## Results

**Figure 3:**
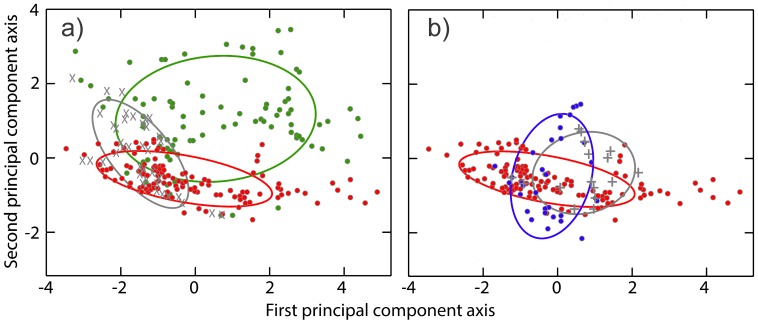
Scatterplot for the first and second axis in a principal component analysis of bioclimatic values. Filled circles represent species with species-specific mtDNA with *T. macedonicus* in green, *T. ivanbureschi* in red and the candidate species in blue. The grey x and+represent *T. macedonicus* and the candidate species with introgressed mtDNA. In (a) the *T. macedonicus* case and in (b) the candidate species case is presented. Ellipses represent mean values plus and minus one standard deviation.

The first and second principal components of the principal component analysis have eigenvalues in excess of unity and together account for 83.5% of the total variance in the data. The third axis and higher contribute marginally to the explained variance and were not further considered. The parameters bio10, bio11 and bio15 load heavily on the first axis and bio16 and bio17 on the second axis. A scatterplot for these two principal components is shown in Fig. 3. Fisher distances including the associated significance levels are reported in Table 1. For the comparison of environmental conditions experienced by species when delineated based upon either mtDNA or nuDNA (hypothesis 1 referred to in the methods), only *T. macedonicus* shows significant differences (P<0.001). For the comparison of environmental conditions in areas with species-specific and introgressed mtDNA within species (hypothesis 2), *T. macedonicus* shows significant differences (P<0.001) whereas the candidate species does not (P>0.05). A comparison of the environmental conditions in the area where species possess species-specific or introgressed mtDNA with those encountered in the range of the mtDNA donor (hypothesis 3) shows that for *T. macedonicus* conditions in the area with introgressed mtDNA are more similar to those in the range of *T. ivanbureschi*, whereas the opposite is found for the candidate species (although Fisher distances are considerably smaller in this case).

**Table 1:** Differences in environmental space as defined from principal component analysis.

Hypothesis tested	Range sections	Fisher distance
1	A+B vs. A	8.217***
1	C vs. B+C+D	1.933 NS
1	D+E vs. E	0.297 NS
2	A vs. B	51.635***
2	D vs. E	1.725 NS
3	A vs. C	116.754***
3	B vs. C	18.888***
3	C vs. D	2.423 NS
3	C vs. E	0.231 NS

The hypotheses tested are numbered as in the main text. Range sections correspond to Fig. 2. NS = non-significant;

*** = significant at p<0.001.

**Figure 4:**
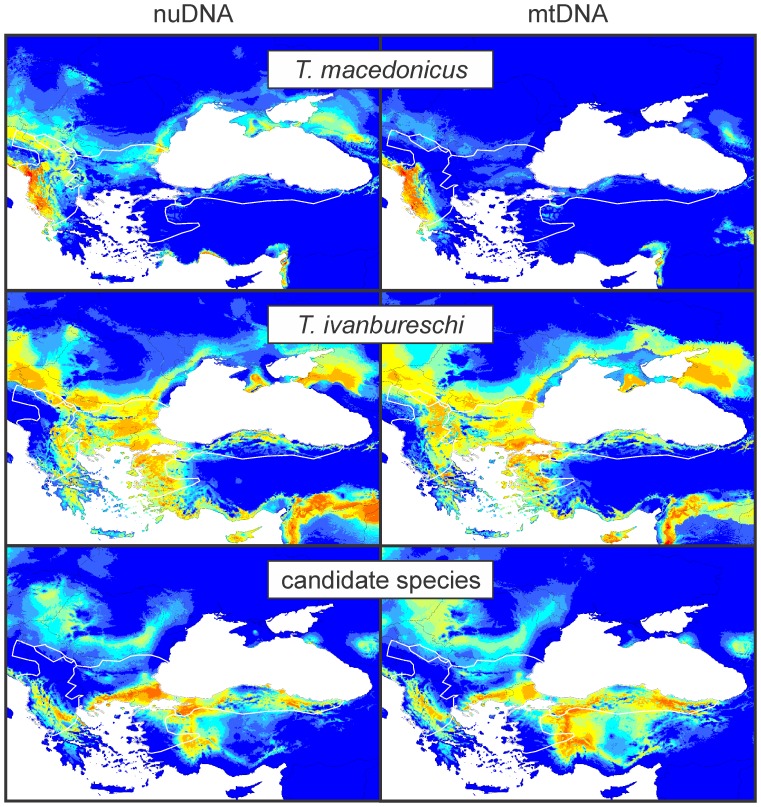
Species distribution models for the three *Triturus* species. Species distribution models as delineated based on nuDNA (left) or mtDNA (right). Species’ ranges are outlined in white lines (cf. Fig. 2).

The six species distribution models are shown in Fig. 4. All models have an AUC support value that is statistically significantly higher than the corresponding null distribution. Comparisons of geographical overlap *D* values are provided in Table 2. For the three pairwise comparisons of sets of *D* values obtained by comparing mtDNA and nuDNA delineated species (hypothesis 4), two are significantly different (*T. macedonicus* vs. the other two species; P<0.001) and one is not (*T. ivanbureschi* vs. the candidate species; P>0.05). For the two pairwise comparisons of the sets of *D* values of the two pairs of species that show mtDNA introgression (hypothesis 5) both show significant differences (P<0.001).

**Table 2:** Differences in the geographical overlap of species distribution models.

Hypothesis tested					Geographical overlap (Schoener’s *D*), based upon	
Range sections compared	C vs. B+C+D	D+E vs. E	A vs. B+C+D	B+C+D vs. E	all locality data	semi-replicates
A vs. A+B	4:***	4:***			0.69	0.67±0.031
C vs. B+C+D		4: NS			0.90	0.88±0.014
E vs. D+E					0.88	0.87±0.032
A+B vs. C			5:***		0.55	0.54±0.023
A vs. B+C+D					0.49	0.44±0.035
C vs. D+E				5:***	0.56	0.57±0.035
B+C+D vs. E					0.57	0.60±0.026

The hypotheses tested are numbered as in the main text. Range sections correspond to Fig. 2. Schoener’s *D* is provided for models based on all locality data and the average and standard deviation (σ) is provided for the hundred semi-replicates based on sub-sampling of locality data. NS = non-significant;

*** = significant at p<0.001.

## Discussion

We study the distribution of species-specific mtDNA and nuDNA in crested newts in SE Europe and adjacent Asia. The three species involved are separated by wide areas in which these genetic markers yield contrasting species identifications (Fig. 2). In both these areas the mtDNA of the central species *T. ivanbureschi* protrudes outside of its range as delineated based on nuDNA, to the west in and around southern Serbia into the range of *T. macedonicus* [36] and to the east in NW Asiatic Turkey into the range of the candidate species [22].

### Environmental Space

For the western case with *T. macedonicus* we find a Fisher distance, reflecting the amount of niche divergence, significantly different from zero, depending on whether the species is delineated by either nuDNA (**A**) or mtDNA (**A+B**) (Table 1, Fig. 3). For the eastern case with the candidate species, this distance (i.e. between **D+E** and **E**) is not significantly different from zero. In other words, using mtDNA as a guidance leads to a flawed estimate of the niche space taken by *T. macedonicus*, but not for the candidate species. For *T. macedonicus* the environmental conditions in the area with foreign mtDNA (range section **B** in Fig. 2) are different from the core of the range (range section **A**) and relatively similar to the environmental conditions experienced by the mtDNA donor *T. ivanbureschi* (range section **C**). Conditions in the region where the candidate species has foreign mtDNA (range section **D**) appear, compared to the core of its range (range section **E**), dissimilar to those in the range of *T. ivanbureschi* (range section **C**), but the difference (between range sections **D** and **E**) is not significantly different from zero.

### Geographical Space

The species distribution models for *T. macedonicus* based on either genetic marker show significantly smaller geographical overlap, and hence more diverged niches, compared to those for *T. ivanbureschi* and the candidate species (Table 2, Fig. 4). In other words, models based on nuDNA and mtDNA are relatively more different for *T. macedonicus* than they are for the candidate species and for *T. ivanbureschi*. Geographical overlap between models for *T. macedonicus* and *T. ivanbureschi* is significantly smaller when localities from the introgression zone are allocated to *T. ivanbureschi* whereas for *T. ivanbureschi* and the candidate species we find the opposite. This suggests that the environmental conditions in the introgression zone of *T. macedonicus* (section **B**) are more similar to those in the range of *T. ivanbureschi* (section **C**) than they are to those in the core of the *T. macedonicus* range (section **A**). Conversely, environmental conditions in the introgression zone of the candidate species (section **D**) are more similar to those in the core of its range (section **E**) than to those in the range of *T. ivanbureschi* (section **C**).

For the western case with *T. macedonicus*, findings are in line with the scenario in Fig. 1c. Here niche divergence is overestimated because the environmental conditions that *T. macedonicus* experiences in the introgression zone, relatively similar to those experienced by mtDNA donor *T. ivanbureschi*, are erroneously attributed to that species. We find this pattern when we analyze the data both in environmental space (by determining Fisher distances in a principal component analysis) and in geographical space (by determining geographical overlap of species distribution models). For the eastern case with the candidate species, findings are in line with the scenario in Fig. 1b, where mtDNA introgression leads to an underestimation of niche divergence because the conditions in the introgression zone, erroneously included in the niche space inhabited by mtDNA donor *T. ivanbureschi*, are actually distinct from that species. However, this result is less clear cut as we only find it for our analysis in geographical space and not for the one in environmental space.

As morphologically cryptic species are often closely related, occasional hybridization is to be expected [37], in which mtDNA introgression is more common and generally acting over wider areas than is nuclear introgression [9], [10]. Hybridization with extensive mtDNA introgression will frequently hamper identification of morphologically cryptic species. In extreme cases the original mtDNA of a species can be completely replaced with that derived from another species [38], [39]. Determining niche divergence between range sections characterized by diverged mtDNA is a promising tool in the discovery of cryptic species [6], [7]. However, we show here that mtDNA introgression can affect estimates of niche divergence. In the two cases we explore we observe underestimation as well as overestimation of niche divergence, under the common course of mtDNA introgression that disturbs the estimates of niche divergence. While we acknowledge the relevance of niche divergence in delineating species (see e.g. the integrated approach in ref. [40]), we also plea for caution, in particular when species ranges are derived from mtDNA alone.

## Supporting Information

Dataset S1 **Locality data partitioned according to nuDNA composition and mtDNA type.**
(XLS)
